# Recognition of Trace Element Contamination Using *Ficus macrophylla* Leaves in Urban Environment

**DOI:** 10.3390/ijerph17030881

**Published:** 2020-01-31

**Authors:** Maria Grazia Alaimo, Daniela Varrica

**Affiliations:** Dipartimento Scienze della Terra e del Mare (DiSTeM), Via Archirafi 22, 90123 Palermo, Italy; mariagrazia.alaimo@unipa.it

**Keywords:** air quality, trace elements, biogeochemistry, *Ficus macrophylla* Desf. ex Pers. leaves, environmental geochemistry

## Abstract

Urban areas are characterized by numerous pollutants emitted by anthropic sources both in the form of solid and gaseous particulates. Biomonitoring is an easy, economical, and accessible approach for the determination of atmospheric pollutants. In this study, we used the leaves of *Ficus macrophylla* Desf. ex Pers., collected in the city of Palermo (Italy), to determine major and trace elements. Geogenic elements exhibited the highest concentrations, making up 99% of the weight of the analyzed elements (Ca, K, Mg, P, S, Na, Fe, and Al); they range 21,400 (Ca) to 122 µg g^−1^ (Al). The remaining elements showed median concentrations in the range 47.5–0.05 µg g^−1^ in the following order of abundance: Sr > Cu > Mn > Zn > Br > Rb > Ba > Pb > Cr > Sb > As > Mo = Sc. Cluster analysis, with Spearman’s coefficient to measure sample similarity, identified five main groups, namely, three clusters related to the geogenic background and marine spray; one cluster linked to elements essential to plants, and a final group attributed to the influence of traffic emissions. Calculated enrichment factors (EF) showed that the enrichments found for P and K were linked to plant metabolism; Na and Mg confirmed the role of sea spray; Cu and Zn underlined the contribution linked to anthropic processes and the role of micronutrients in plants.. As, Cr, and Mo had EF values ranging from 10 and 20, and Sb had EF > 90. From geochemical distribution maps of As, Cr, Mo, and Sb it was observed that metal and metalloid concentrations were higher in urban areas and immediately decreased as one moved away from these areas. Local pollution sources play a great role in trace element concentrations in airborne particulate matter. The present study confirms that *Ficus macrophylla* leaves are suitable for screening an urban environment to identify concentrations of inorganic chemicals, since they have high tolerance to pollution.

## 1. Introduction

Urban areas are characterized by numerous pollutants emitted by anthropic sources in the form of solid and gaseous particulates [1]. Primary emission sources of anthropogenic air pollutants are car traffic, industrial activities, power plants, and domestic fuel [2,3]. Trace elements released into the atmosphere from various anthropogenic sources pose a threat to human health [4,5] with acute and chronic effects, affecting many different systems and organs [6]. The main manifestations concern diseases of the respiratory (allergies, asthma, and lung emphysema [7]) and cardiovascular systems [8,9], sometimes even degenerating into lung cancer [10,11]. In recent years, biomonitoring has received growing favor from the scientific community because it is useful in environmental control programs. Bioaccumulators (1) reflect the elemental ambient conditions; (2) offer a practical way of studying the spatial distribution of airborne contaminants; (3) provide a measure of integrated exposure over a certain period time; (4) are an easy, economical alternative to air sampling filters; (5) allow a higher density of sampling sites; and (6) allow an accessible approach for the analytical determination of trace elements [12,13,14]. 

Several studies have used lower plants, mosses [15,16,17], lichens [18,19,20,21], and parts of higher plants such as leaves as passive samplers in different areas contaminated by natural and anthropic sources [13,22,23,24].

Plant leaves are widely used due to their ability to absorb pollutants through stomata and cuticles or indirectly through the roots after the deposition of atmospheric pollutants in the soil [25]. The ability to absorb trace elements through dry or wet deposition depends on leaf surface morphology, which regulates its absorption [26,27,28,29]. Some researchers, for example, have used *Nerium Oleander* L. leaves in environmental geochemical studies to document the presence of trace elements of anthropogenic origin in airborne particulate matter of urban areas [3,23,30,31]. Pine needles have been used to reveal the presence of metal concentrations in the environment, in urban areas [22,32,33,34], in industrial areas [24,35] and in mining areas [36]. Only a few studies have concerned the use of the genus *Ficus* for environmental monitoring; in fact, the determination of the content of metals and metalloids has been made on leaves of *Ficus Benjamin* [37] and *Ficus microcarpa* [38] in urban environments.

In this study the leaves of *Ficus macrophylla* Desf. ex Pers. have been chosen to evaluate air quality in the city of Palermo, as there are no monitoring studies in the literature that use this species of *Ficus* as a biomonitor. This tree species is an evergreen plant which is very common in gardens and along urban avenues, and the morphological structure of leaves could facilitate a dust-retaining capability. The main objective was to determine trace element concentrations, build spatial distribution maps of the metals and metalloids, and identify their local sources.

## 2. Materials and Methods

### 2.1. Study-Area Description 

**Figure 1:**
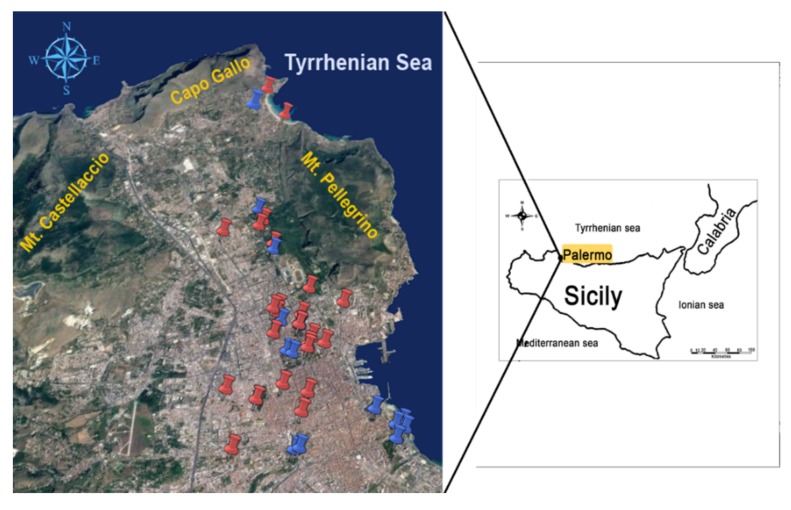
Study area in relation to sampling site location of *Ficus macrophylla*. Red indicates samples collected from the long main urban road; blue indicates samples collected from city gardens.

Palermo is the largest urban area of Sicily, having about 680,000 inhabitants and a metropolitan area populated by more than 1 million people. The city is situated on the north-western coast of the island, bordered on the northeast by the Tyrrhenian Sea and surrounded by mountains (Monti di Palermo) reaching 500–1000 m above sea level (Figure 1); the study area is entirely covered by sedimentary rocks (limestone, clay, marly-clay, and white or yellow quaternary biocalcarenite; most of the eastern sector of the Piana di Palermo is covered by a bright red-colored soil rich in Fe-oxides, known as “Terra Rossa”) [39,40]. The climate of Palermo is typically Mediterranean, with hot summers and temperate winters. Winds during the autumn months, generally breezes, predominantly originate from the WNW and WSW sectors. 

### 2.2. General Species Characteristics 

*Ficus macrophylla* Desf. ex Pers is a species belonging to the genus *Ficus* Linn. (*Moraceae, Magnoliophita*). The tree has a large crown composed of branches and shiny and evergreen leaves. It contains a set of roots of various caliber and shape which extend vertically up to the ground with the function of supporting the crown and supplying water to the leaves [41,42]. Usually, adult plants have an average height of 25 m and a tree-trunk circumference of about 18 m. It is widespread in the city gardens and along major roads of Palermo. The plants arrived in Palermo from Australia at the end of the 17th century and grew in surprising proportions and shapes. The widespread presence of exceptional examples of *Ficus macrophylla* in Palermo has great aesthetic value for the city [43].

### 2.3. Sampling and Analytical Method

A total of 39 composite samples of several years of leaf growth from *Ficus macrophylla* Desf. ex Pers were collected in October 2018 (Figure 1). The sampling sites were chosen according to the distribution of all the monumental specimens recorded in the city of Palermo [44].

In Table 1 we report the location and description of sampling sites. Each sample, made up of several subsamples collected from all over the tree, was stored in a paper bag. The unwashed samples were dried for 48 h at 40 °C and then ground to a fine powder. A portion of each sample was analyzed for major and trace elements using instrumental neutron activation analysis (INAA) and inductively coupled plasma optical emission spectroscopy (ICP-OES) (Br, Ca, K, Mg, P, Na, and Sc) at Activation Laboratories Ltd. (Ontario, Canada) using NBS 1572 and 1632B as the standard reference material. The 13 trace elements (Al, As, Ba, Cr, Cu, Fe, Mn, Mo, Pb, Rb, Sb, Sr, and Zn) were determined at Dept. Scienze della Terra e del Mare, University of Palermo, using an inductively coupled plasma mass spectrometer (Elan 6100 DRC-e, PerkinElmer) after the addition of Re–Sc–Y as internal standards. For As, Cr, and Fe, the ICP-MS was operated in dynamic reaction cell (DRC) mode with CH_4_ as the reaction gas. All standard solutions were prepared with 18 MΩ cm demineralized water, ICP Multielement Standard Solutions XXI CertiPUR (Merck), and Mo and Sb CertiPUR standards (Merck). Calibration curves ranging 0.05 to 500 μg L^−1^ were constructed. To minimize matrix effects, the standard addition technique was used for all metal determinations. Sample blanks were also analyzed, and the operational limit of detection (LOD) for each element was calculated as three times the standard deviation of the analyte concentration in the blank samples. In order to validate the analytical procedure, standard reference material NIST SRM 1515 Apple Leaves was analyzed for the corresponding elements. Metal recovery rates resulted in good agreement with the certified concentrations, ranging between 89% and 107%. 

Maps were generated using the Surfer Software edited by Golden Software Inc. (Golden, Colorado, USA). The selected gridding method was Kriging [45].

Data were statistically analyzed with the STATISTICA program [46]. All tests in this study were considered significant at *p* < 0.01. The Kolmogorov–Smirnov test, with a level of significance set at *p* < 0.01, was used to verify the normality of the data distribution. The non-parametric Mann–Whitney test at *p* < 0.05 was also used to verify the statistical significance of observed differences between sampling sites.

## 3. Results and Discussion

Table 2 shows the chemical compositions of major and trace elements determined in *Ficus macrophylla* leaf samples on a dry-weight basis; we also report the mean and standard deviation of samples broken into two groups. “Urb” is representative of samples collected from the long main urban road and “CG” of samples taken in the main city gardens.

Most of the analyzed elements, on the basis of the kurtosis and skewness coefficient, showed a leptokurtic distribution with a skew to the right. Ca, Mg, S, As, Cr, Mn, and Sr showed a platykurtic distribution with a skewed to the right, and only K, Ba, Rb, and Zn showed a platykurtic distribution with a skew to the left. The application of the Kolmogorov–Smirnov test confirmed that the data had asymmetric distribution. Typical geogenic elements exhibited the highest concentrations and have been reported in the order of decreasing concentration; other elements are listed in alphabetical order. Major elements generally made up 99% of the weight of the analyzed elements (Ca, K, Mg, P, S, Na, Al, and Fe) and ranged 21,400 (Ca) to 122 µg g^−1^ (Al). The great predominance of these elements is not surprising: firstly, Ca, Mg, Fe, Al, and Na, in general, are typical elements linked to the resuspension of soil material and sea spray, and these processes are considered fundamental for the formation of airborne particulate matter, as reported by several studies conducted in Palermo city [22,30,47,48]; secondly, K, P, and S are macronutrients and play an important role in plant growth. The remaining elements showed median concentrations in the range 47.5–0.05 µg g^−1^ and had the following order of abundance: Sr > Cu > Mn > Zn > Br > Rb > Ba > Pb > Cr > Sb > As > Mo = Sc. In Table 3 we report Spearman’s rank correlation matrix (*p* < 0.01; *ρ* = 0.37). 

Some elements showed a statistically significant positive correlation, indicating a common origin. Correlations between elements were found to suggest a multitude of sources that may contribute to a load of trace elements in *Ficus macrophylla* leaves. 

**Figure 2:**
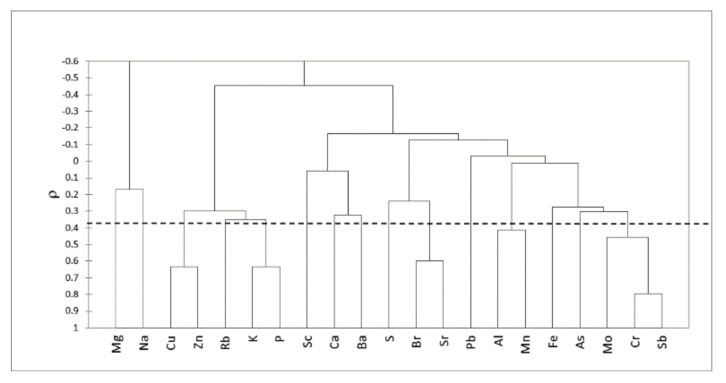
Cluster analysis dendrogram for 39 samples and 21 elements. Cluster analysis was based on Spearman’s rank correlation *ρ*.

A useful method employed to simplify the complex data set, with the aim of identifying relationships between variables and possible sources of air pollution, is cluster analysis (CA). The clustering procedure was performed with Spearman’s coefficient as a measure of sample similarity. Results are displayed in the bidimensional hierarchical diagram of Figure 2. Five main groups of related elements may be identified at *ρ* < 0.37 (*p* < 0.01). Lead is not linked to any of the identified groups. Two clusters are related to the geogenic background: Ba and Ca are representative of Palermo’s calcareous substrate, and the second cluster, comprising Mn and Al, is associated with the clay soil component [22,39]. Bromine and strontium are associated with sea spray [19,22,49,50]. The group comprising Cu, Zn, Rb, K, and P represents the essential elements for plants [51].

The last cluster, comprising Cr, Mo, and Sb, can be attributed to the influence of traffic on air pollution. The elements identified by CA as geogenic were observed in other biomonitoring studies in Palermo. Several works have reported the fundamental role of calcarenite bedrocks and the local “terra rossa” soil in indicating the crustal origin [22,40,47] of the elements deposited on leaves. Likewise, sea spray has an important contribution to particulate matter concentrations in Palermo. Dongarrà et al. [48] have found that NaCl concentrations in Palermo constitute 7.4% of PM_2.5_ and 11.2% of PM_10_ mass levels. 

**Figure 3:**
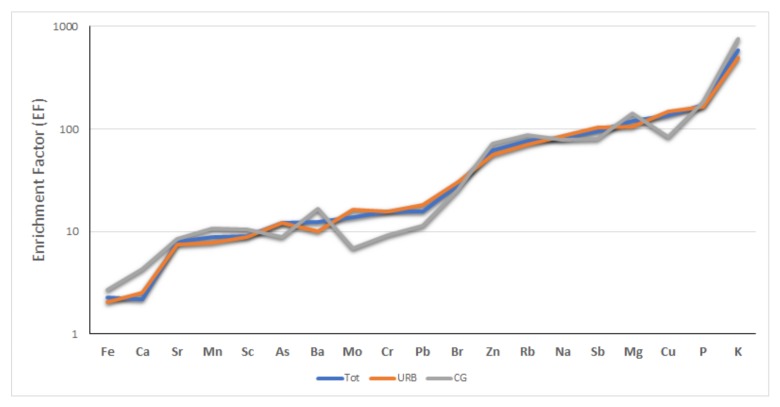
Average enrichment factors (EFs) for the analyzed elements in *Ficus macrophylla* leaves.

To confirm the anthropogenic source of the groups defined from cluster analysis, we calculated the enrichment factors (EF). EFs were calculated by dividing their relative abundance in *Ficus macrophylla* leaves by their relative average abundance in the local soil: EF = (X/Al)_FL_/(X/Al)_LS_. According to Varrica et al. [34], the average local soil (LS) is considered to be made up of carbonate rocks (80%), clay minerals (10%), and “terra rossa” soil (10%). Aluminum was selected as the reference element (Figure 3).

The enrichments found for P and K are linked to plant metabolism, and Na and Mg confirm the role of sea spray. The EF of Cu and Zn underline their contribution linked to both anthropic processes and the role of micronutrients in plants.. Arsenic, Cr, Mo, and Sb were found to have EF values ranging from 10 to 100 with respect to the local soil, indicating a source link to vehicular traffic. Other trace elements (Mn, Sr, Ca, and Fe) showed EF < 10. The same enriched elements were found in particulate matter PM_2.5_ [48]. We interpret these findings as clear evidence that these metals and metalloids originated from a common source identified in general with gaseous emissions and the mechanical part deterioration of motor vehicles. Arsenic is derived from oil combustion; antimony, chromium, and molybdenum appear to be associated with non-exhaust vehicle emissions, including release by the mechanical abrasion of metal structures of vehicles, engine components, tires, and brake linings [48,51,52,53,54,55,56]. In addition, Sb concentration in particulate matter has appeared to act as a fingerprinting tool in identifying the contribution of road vehicles to traffic-derived particulate matter [48,56,57]. In the past, brake pads were made with asbestos. During the end of the 20th century, with the elimination of asbestos from the market, pads began to be produced by other materials, including fillers such as Sb sulphates, kaolinite clays, Mg and Cr oxides, and metal powders. A study by Rossini Oliva and Rautio [38] on *Ficus microcarpa* L. leaves growing in Palermo has highlighted the role of vehicular traffic pollution in the contents of some trace elements (Cr, Pb, Ba, and Cu) and the decreased content of lead observed over several years in relation to the increase in unleaded petrol use.

**Figure 4:**
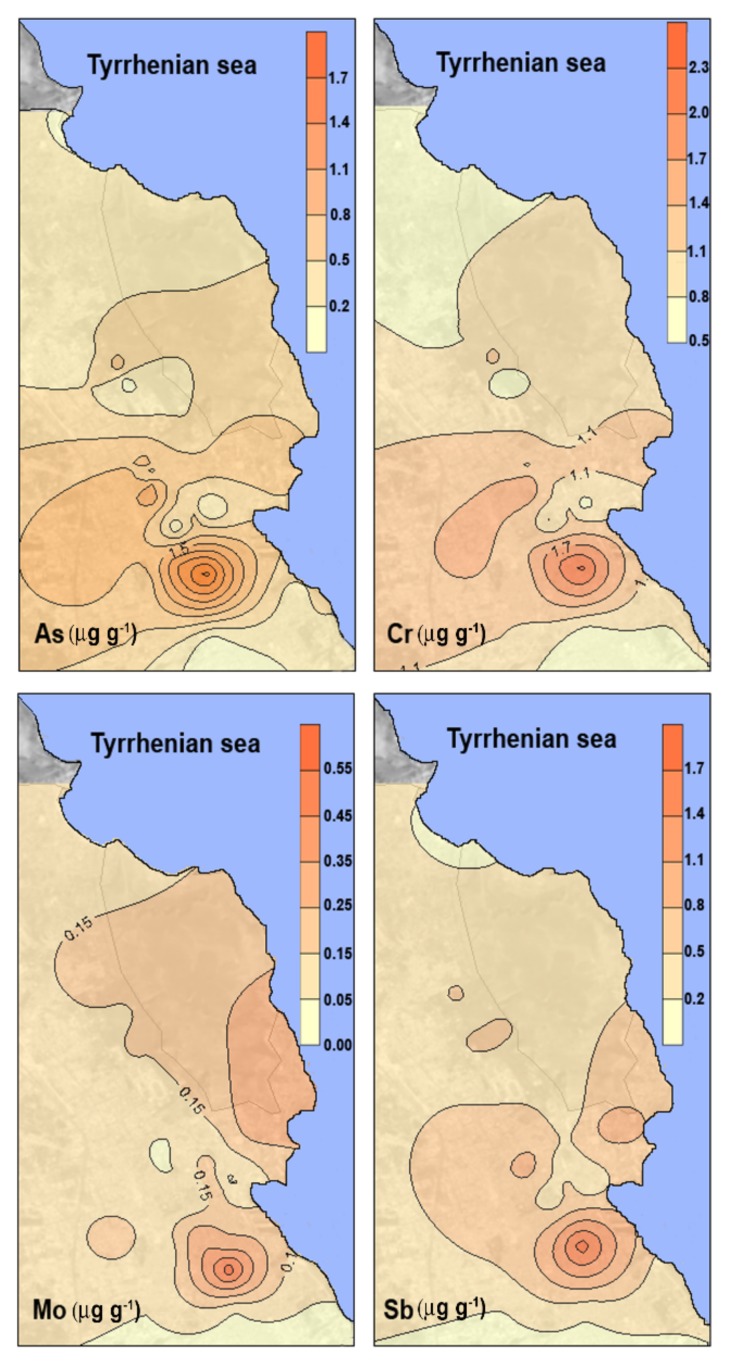
Areal distribution maps of As, Cr, Mo, and Sb in *F. macrophylla* leaves. Data are expressed in μg g^−1^ (dry weight).

The distribution maps of As, Cr, Mo, and Sb, shown in Figure 4, highlight the anthropogenic geochemical anomalies within the study area. Metal and metalloid concentrations are higher in urban areas and immediately decrease as one moves away from these areas, indicating that local pollution sources play a great role in metal and metalloid concentrations in airborne particulate matter.

Elemental concentrations of trace elements in *Ficus macrophylla* leaves were statistically compared, taking the sampling site into account, using the non-parametric Mann–Whitney test with a significance level of *p* < 0.05. Four out of 13 elements exhibited significant differences between the two groups (Table 4). 

**Figure 5:**
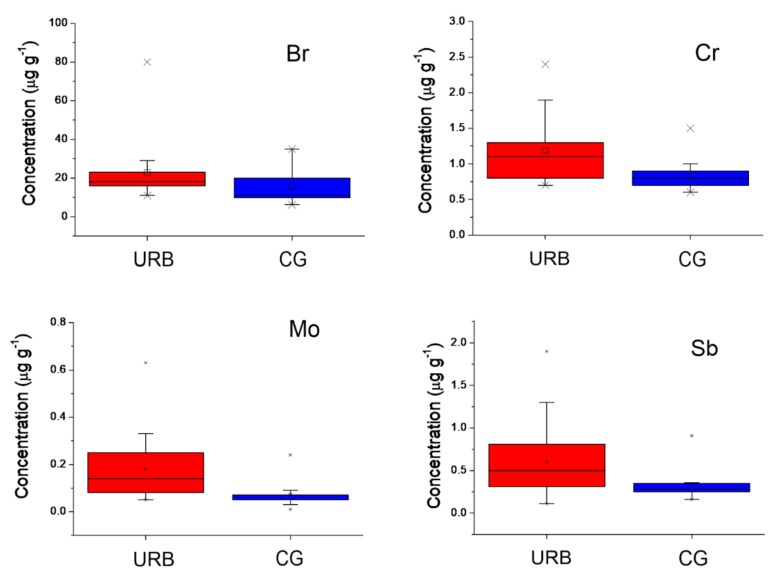
Box and whisker plots displaying medians, quartiles, and extremes of Br, Cr, Mo, and Sb concentrations in *Ficus macrophylla* samples. Box interquartile ranges (25–75%) with median indication are shown by solid lines; vertical lines outside the box (whiskers) indicate the range (1–99%) between the highest and lowest observations, excluding the minimum and maximum. Data are given in μg g^−1^ (dry biomass).

Br, Cr, Mo, and Sb had much higher concentrations in urban sites than in city gardens, as shown in Figure 5.

### Effect of As, Cr, Mo, and Sb on Human Health

Many trace elements are nutritionally important for human growth, but “All things are poison and nothing is without poison. Solely the dose determines that a thing is not a poison” (*Omnia venenum sunt: nec sine veneno quicquam existit. Dosis sola facit, ut venenum non fit*, Paracelsus, 1493–1541).

Air pollutants can have different effects on human health. The dangerousness and possible toxicity of some elements can be caused by different factors, including composition, dose, and time of exposure [11]. The human body comes into contact with trace elements through different paths that can be grouped into three broad categories: respiration, ingestion of food and water, and absorption through the skin.

The toxic effects of trace elements, apart from their inducing oxidative stress, can also be attributed to their ability to substitute diverse polyvalent cations (calcium, zinc, and magnesium). Metals accumulate in cellular organelles binding to proteins [58] and inhibiting a large number of enzymes [59].

Arsenic is a naturally occurring element that is widely distributed in the Earth’s crust. Arsenic is classified chemically as a metalloid. Arsenic exposure might cause, in addition to nucleic damage, disruption of mitochondrial and ribosomal function as well as modulation of proliferative and inflammatory pathways [60]. It is also a potent enzyme inhibitors [61,62]. Inorganic arsenic is a confirmed carcinogen that can also cause cardiovascular, respiratory, gastrointestinal, and neurological problems. Long-term exposure to high levels of inorganic arsenic can lead to local irritation and dermatitis [63].

Chromium is a transition element and has a different effect on human health depending on its oxidation state (Cr III or VI). Hexavalent chromium is toxic and carcinogenic; its toxicity derives from its ability to diffuse through cell membranes and oxidize biological molecules [64,65]. The main health problems caused by chromium are bronchial asthma, ulcers, lung and nasal tumors, skin allergies, and reproductive and developmental problems. Trivalent chromium, on the other hand, is nontoxic and an essential nutrient.

Molybdenum exists in several valence states. It can interact with the enzymatic system. Though an essential element in tiny amounts, it can be highly toxic in large doses. Exposure to too much molybdenum dust can manifest itself through systemic, immunological, neurological, and carcinogenic effects on human health [66,67]. 

Antimony toxicity depends on its chemical state; it does not cause strong ecotoxicological effects but can be mutagenic. Trivalent compounds have toxic effects that are about ten times greater than those of pentavalent species binding to plasma cells [68]. Its primary toxicity targets are the heart, gastrointestinal tract, musculoskeletal system, liver, and pancreas [69].

## 4. Conclusions

In this work, the leaves of *Ficus macrophylla* Desf. ex Pers. were used as biomonitors to assess air pollution in the urban environment. Sufficient evidence was provided which demonstrated the accumulation of metals and metalloids in the leaves of F. *macrophylla* collected in different Palermo sites. This sampling method is highly recommended as an economical and accessible means of detecting and monitoring inorganic pollutants in atmospheric dust. These data revealed the multitude of sources that contribute to the atmospheric particulate matter of the city of Palermo, ranging from anthropogenic sources linked exclusively to gaseous emissions and the deterioration of the mechanical parts of motor vehicles, to geogenic sources that include both sea spray and the resuspension of soil dust. 

Vehicular emissions, known to be dangerous to human health, also have a negative impact on the life cycle of plants. 

The present study has confirmed that *Ficus macrophylla* Desf. ex Pers. leaves are suitable for screening an urban environment as they have a high tolerance to pollutants. The introduction of these plants in urban areas is desirable, as they function as biofilters of atmospheric pollution, accumulating toxic metals in their leaf and root systems, and making the environment less toxic.

## Figures and Tables

**Table 1:** Sampling sites and location descriptions. Legend: Urb, samples collected from the long main urban road; CG, samples collected from city gardens.

Samples	Location	Characteristics
FC1	Urb	urban road exposed to heavy traffic, composed of cars and urban buses
FC2	Urb	urban road characterized by lower traffic density
FC3	Urb	large square exposed to traffic mainly composed of cars and urban buses
FC4	Urb	urban road characterized by lower traffic density
FC5	Urb	urban road characterized by lower traffic density
FC6	Urb	urban road characterized by lower traffic density
FC7	Urb	urban road characterized by lower traffic density
FC8	Urb	urban road exposed to medium amount of traffic of cars
FC9	Urb	urban road exposed to medium amount of traffic of cars
FC10	Urb	urban road exposed to medium amount of traffic of cars
FC11	Urb	urban road exposed to medium amount of traffic of cars
FC12	Urb	urban road exposed to heavy traffic, composed of cars, heavy-duty vehicles and urban and extra-urban buses
FC13	Urb	urban road exposed to heavy traffic, composed of cars and urban buses
FC14	Urb	urban road exposed to high traffic flow, composed of cars, heavy-duty vehicles and urban and extra-urban buses
FC15	Urb	urban road exposed to heavy traffic, composed of cars and urban and extra-urban buses
FC16	Urb	urban road exposed to medium amount of traffic of cars
FC17	Urb	urban road exposed to medium amount of traffic of cars
FC18	Urb	urban road exposed to high traffic flow, composed of cars, heavy-duty vehicles and urban buses
FC19	Urb	urban road exposed to medium amount of traffic of cars
FC20	Urb	urban road exposed to high traffic flow, composed of cars, heavy-duty vehicles and urban and extra-urban buses
FC21	Urb	a little square in front of the railway station, exposed to heavy traffic, composed of cars and urban and extra-urban buses
FC22	Urb	urban road exposed to heavy traffic, composed of cars and urban buses
FC23	Urb	large square in front of the sea exposed to heavy traffic by cars, urban and extra-urban buses
FC24	Urb	urban road exposed to heavy traffic composed of cars, urban and extra-urban buses
FC25	Urb	urban road exposed to high traffic flow, composed of cars, heavy-duty vehicles and urban and extra-urban buses
FC26	CG	urban garden without any direct influence of vehicular traffic
FC27	CG	urban garden without any direct influence of vehicular traffic
FC28	CG	urban garden without any direct influence of vehicular traffic
FC29	CG	urban garden without any direct influence of vehicular traffic
FC30	CG	urban garden without any direct influence of vehicular traffic
FC31	CG	urban garden without any direct influence of vehicular traffic
FC32	CG	urban garden without any direct influence of vehicular traffic
FC33	CG	urban garden without any direct influence of vehicular traffic
FC34	CG	urban garden without any direct influence of vehicular traffic
FC35	CG	urban garden without any direct influence of vehicular traffic
FC36	CG	urban garden without any direct influence of vehicular traffic
FC37	CG	urban garden without any direct influence of vehicular traffic
FC38	CG	urban garden without any direct influence of vehicular traffic
FC39	CG	urban garden without any direct influence of vehicular traffic

**Table 2:** Basic statistical parameters for a total of 39 *Ficus macrophylla* leaf samples. Data given in μg g^−1^ (dry biomass). Legend: N, number of analyzed samples; Std, standard deviation; MAD, median absolute deviation.

	N	Mean ± Std	Median	Minimum	Maximum	Skewness	Kurtosis	MAD	N	URB	N	CG
Ca	39	21,506 ± 4195	21,400	9850	30,350	−0.27	0.85	2700	25	21,748 ± 3733	14	21,075 ± 5039
K	39	17,897 ± 4134	17,800	10,100	26,700	0.19	−0.64	3150	25	17,220 ± 3582	14	19,107 ± 4879
Mg	39	8239 ± 1496	7900	5700	12,000	0.64	0.10	800	25	8238 ± 1470	14	8242 ± 1597
P	39	1602 ± 341	1580	162	2470	−1.25	8.82	130	25	1612 ± 247	14	1585 ± 477
S	39	1253 ± 177	1240	860	1730	0.43	0.44	120	25	1288 ± 178	14	1190 ± 162
Na	39	808 ± 1509	459	265	9423	5.31	29.79	116	25	862 ± 1794	14	714 ± 836
Fe	39	230 ± 199	175	10	880	2.17	4.26	55.0	25	254 ± 203	14	189 ± 192
Al	39	261 ± 401	122	65	2500	4.88	26.7	23.0	25	320 ± 490	14	157 ± 103
As	37	0.17 ± 0.11	0.16	0.03	0.46	0.97	0.75	0.06	25	0.19 ± 0.11	14	0.14 ± 0.11
Ba	37	9.00 ± 3.21	9.00	5.00	15.0	0.22	−0.99	3.00	25	8.41 ± 2.99	14	10.1 ± 3.43
Br	37	20.4 ± 12.9	17.0	6.30	80.0	2.98	12.1	6.00	25	22.8 ± 14.2	14	15.7 ± 8.94
Cr	37	1.06 ± 0.40	0.90	0.60	2.40	1.40	2.16	0.20	25	1.19 ± 0.42	14	0.82 ± 0.24
Cu	39	26.9 ± 15.0	23.0	16.0	99.0	3.80	15.6	3.00	25	26.5 ± 11.0	14	27.8 ± 20.8
Mo	37	0.14 ± 0.12	0.09	0.02	0.63	1.93	5.21	0.04	25	0.18 ± 0.13	14	0.07 ± 0.05
Mn	39	22.9 ± 6.77	22.0	14.0	45.0	1.09	1.52	5.00	25	23.4 ± 6.11	14	22.1 ± 7.98
Pb	39	2.61 ± 1.32	2.65	0.49	8.00	1.76	6.13	0.75	25	3.01 ± 1.39	14	1.89 ± 0.84
Rb	37	9.86 ± 3.78	10.0	4.00	17.0	0.26	−0.92	3.00	25	9.87 ± 3.73	14	9.84 ± 4.03
Sb	37	0.51 ± 0.36	0.40	0.11	1.90	1.91	4.80	0.17	25	0.61 ± 0.40	14	0.34 ± 0.19
Sc	37	0.05 ± 0.01	0.05	0.02	0.08	−0.98	3.29	0.01	25	0.05 ± 0.005	14	0.04 ± 0.01
Sr	39	46.1 ± 12.6	47.5	15.5	75.5	−0.05	0.20	6.50	25	48.7 ± 12.7	14	41.3 ± 11.5
Zn	39	21.0 ± 4.09	21.0	14.5	31.0	0.43	−0.13	3.00	25	21.5 ± 4.01	14	20.1 ± 4.21

**Table 3:** Correlation matrix for selected elements (*p* < 0.01; *ρ* = 0.37).

Spearman Matrix Correlation																					
	Al	Ca	Fe	K	Mg	Na	P	S	As	Ba	Br	Cr	Cu	Mo	Mn	Pb	Rb	Sb	Sc	Sr	Zn
**Al**	**1.00**	0.13	0.25	−0.17	−0.05	0.17	−0.12	0.10	0.05	−0.13	−0.08	0.14	−0.02	0.11	**0.41**	−0.03	−0.12	0.06	0.04	−0.12	−0.15
**Ca**		**1.00**	0.06	−0.45	−0.07	0.10	−0.29	−0.07	−0.12	0.32	0.05	0.00	−0.43	0.05	0.29	0.18	−0.35	0.13	0.09	0.29	−0.40
**Fe**			**1.00**	−0.06	−0.16	−0.03	0.15	0.11	0.29	0.02	−0.01	**0.54**	0.10	0.28	0.31	0.16	−0.01	0.35	0.06	−0.12	0.11
**K**				**1.00**	−0.22	−0.25	**0.63**	−0.19	0.23	0.06	−0.21	0.08	0.36	0.07	−0.20	−0.15	**0.51**	0.03	0.06	−0.38	0.33
**Mg**					**1.00**	0.17	−0.24	−0.09	−0.19	−0.18	−0.21	−0.35	0.09	−0.18	0.01	−0.03	0.17	−0.25	0.01	−0.14	0.12
**Na**						**1.00**	−0.47	−0.05	−0.69	−0.13	0.28	−0.07	−0.10	−0.15	0.10	−0.06	−0.04	−0.05	−0.09	0.01	−0.25
**P**							**1.00**	0.16	**0.59**	0.24	−0.01	0.29	0.31	0.07	−0.06	−0.23	0.35	0.15	0.15	−0.23	0.41
**S**								**1.00**	0.08	−0.04	**0.37**	0.21	0.10	0.22	0.36	0.01	−0.41	0.16	−0.17	0.24	0.24
**As**									**1.00**	0.02	−0.07	**0.37**	0.08	0.30	0.01	0.18	0.17	0.32	0.19	−0.13	0.36
**Ba**										**1.00**	0.10	0.01	0.04	−0.06	0.02	−0.15	−0.22	0.12	0.06	0.07	0.05
**Br**											**1.00**	**0.38**	0.06	0.22	0.24	−0.06	−0.06	**0.52**	−0.06	**0.60**	0.20
**Cr**												**1.00**	0.21	**0.46**	0.16	0.17	0.06	**0.79**	0.00	0.16	0.27
**Cu**													**1.00**	0.20	−0.11	0.01	0.35	0.20	−0.05	0.02	**0.64**
**Mo**														**1.00**	0.16	0.15	−0.05	**0.50**	0.23	0.03	0.25
**Mn**															**1.00**	0.03	−0.32	0.09	0.17	0.15	0.15
**Pb**																**1.00**	−0.09	−0.01	−0.13	0.16	0.17
**Rb**																	**1.00**	0.04	0.08	−0.26	0.30
**Sb**																		**1.00**	0.02	0.32	0.27
**Sc**																			**1.00**	−0.15	−0.07
**Sr**																				**1.00**	0.01
**Zn**																					**1.00**

**Table 4:** Non-parametric Mann–Whitney test between leaf samples from urban and city-garden sites (*p* < 0.05).

	N. URB	N. CG	*p* Level
**As**	24	13	0.11
**Ba**	24	13	0.15
**Br**	24	13	**0.03**
**Cr**	24	13	**0.003**
**Cu**	25	14	0.38
**Mo**	24	13	**0.03**
**Mn**	25	14	0.32
**Pb**	25	14	0.22
**Rb**	24	13	1.00
**Sb**	24	13	**0.01**
**Sc**	24	13	0.81
**Sr**	25	14	0.07
**Zn**	25	14	0.27
